# Supplementary material for: Molecular Characterizations of the er1 Alleles Conferring Resistance to Erysiphe pisi in Three Chinese Pea (Pisum sativum L.) Landraces
Source: Int J Mol Sci. 2022 Oct 10;23(19):12016. doi: 10.3390/ijms231912016 (PMC9569905; doi:10.3390/ijms231912016)
Supplement: Supplementary file 1 [file ijms-23-12016-s001.zip › ijms-1879841-supplementary.pdf]

Table S1. Detection of functional markers of 56 pea germplasms with known phenotype and carrying known resistance genes (*er1* alleles), and the six parents resistant/susceptible to *E. pisi*, using *KASPar-er1*-13 and *KASPar-er1*-14 specific for *er1*-13 and *er1*-14 at the *er1* locus, respectively.

No.	Accession No./ Germplasm name	Origin	Phenotype	*er1* allele	*er1*-13 genotype	*er1*-14 genotype	Reference
1	Bawan 6	China, Hebei	S	Er1	T:T	T:T	[15]
2	Longwan 1	China, Gansu	S	Er1	T:T	T:T	[40]
3	Chenwan 8	China, Sichuan	S	Er1	T:T	T:T	This study
4	Suoshadabaiwan	China, Chongqing	I	er1-4	T:T	T:T	This study
5	Dabaiwandou	China, Yunnan	I	er1-13	-:-	T:T	This study
6	Guiwan 1	China, Guangxi	I	er1-14	T:T	-:-	This study
7	G0004382	Australia	I	er1-1	T:T	T:T	[32]
8	G0003925	Canada	I	er1-1	T:T	T:T	[39]
9	Cooper	Canada	I	er1-1	T:T	T:T	[39]
10	20012	China, Gansu	I	er1-1	T:T	T:T	[32]
11	L0314	China, Yunnan	I	er1-1	T:T	T:T	[40]
12	G0003942	Unknown country	I	er1-1	T:T	T:T	[32]
13	G0003943	Unknown country	I	er1-1	T:T	T:T	[32]
14	G0004417	Australia	I	er1-2	T:T	T:T	[32]
15	G0004434	Australia	I	er1-2	T:T	T:T	[32]
16	G0004448	Australia	I	er1-2	T:T	T:T	[32]
17	G0004450	Australia	I	er1-2	T:T	T:T	[32]
18	G0006273	China, Gansu	I	er1-2	T:T	T:T	[15]
19	Xucai1	China, Hebei	I	er1-2	T:T	T:T	[15]
20	G0002848	Denmark	I	er1-2	T:T	T:T	[32]
21	G0002971	England	I	er1-2	T:T	T:T	[32]
22	G0002859	Germany	I	er1-2	T:T	T:T	[32]
23	G0002860	Germany	I	er1-2	T:T	T:T	[32]
24	G0002883	Germany	I	er1-2	T:T	T:T	[32]
25	G0003897	ICRISAT	I	er1-2	T:T	T:T	[32]
26	G0003907	ICRISAT	I	er1-2	T:T	T:T	[32]
27	G0003911	ICRISAT	I	er1-2	T:T	T:T	[32]
28	G0003961	India	I	er1-2	T:T	T:T	[32]
29	G0006285	Japan	R	er1-2	T:T	T:T	[32]

30	G0002128	USA	I	er1-2	T:T	T:T	[32]
31	G0002129	USA	I	er1-2	T:T	T:T	[32]
32	G0006514	Canada	R	er1-2	T:T	T:T	[32]
33	G0006515	Canada	R	er1-2	T:T	T:T	[32]
34	G0006516	Canada	I	er1-2	T:T	T:T	[32]
35	G0006519	Canada	I	er1-2	T:T	T:T	[32]
36	G0005576	China, Chongqing	I	er1-2	T:T	T:T	[18]
37	Jia2	China, Gansu	I	er1-2	T:T	T:T	[32]
38	Texuan11	China, Gansu	I	er1-2	T:T	T:T	[32]
39	L1332	China, Yunnan	I	er1-2	T:T	T:T	[40]
40	L1335	China, Yunnan	I	er1-2	T:T	T:T	[40]
41	Yunwan18	China, Yunnan	R	er1-2	T:T	T:T	[32]
42	Yunwan35	China, Yunnan	I	er1-2	T:T	T:T	[32]
43	L2157	China, Yunnan	I	er1-2	T:T	T:T	[32]
44	PI391630	China, Guangdong	I	er1-4	T:T	T:T	[9]
45	G0002102	Canada	I	er1-6	T:T	T:T	[32]
46	G0003694	China, Hebei	R	er1-6	T:T	T:T	[18]
47	G0001747	China, Yunnan	R	er1-6	T:T	T:T	[32]
48	G0001752	China, Yunnan	I	er1-6	T:T	T:T	[18]
49	G0001763	China, Yunnan	I	er1-6	T:T	T:T	[18]
50	G0001764	China, Yunnan	I	er1-6	T:T	T:T	[18]
51	G0001767	China, Yunnan	I	er1-6	T:T	T:T	[18]
52	G0001768	China, Yunnan	I	er1-6	T:T	T:T	[18]
53	G0001773	China, Yunnan	I	er1-6	T:T	T:T	[32]
54	G0003974	China, Yunnan	I	er1-7	T:T	T:T	[32]
55	G0003975	China, Yunnan	I	er1-7	T:T	T:T	[32]
56	G0003895	ICRISAT	I	er*1*-7	T:T	T:T	[17]
57	G0003899	ICRISAT	I	er1-7	T:T	T:T	[17]
58	G0003967	India	I	er1-7	T:T	T:T	[17]
59	G0004394	Nepal	R	er1-7	T:T	T:T	[17]
60	G0003958	India	I	er1-7	T:T	T:T	[17]
61	G0004389	Afghanistan	I	er1-8	T:T	T:T	[32]
62	G0004400	Australia	I	er1-9	T:T	T:T	[32]
